# The impact of living with long‐term conditions in young adulthood on mental health and identity: What can help?

**DOI:** 10.1111/hex.12944

**Published:** 2019-07-25

**Authors:** Ceri Wilson, Jennifer Stock

**Affiliations:** ^1^ Faculty of Health, Education, Medicine and Social Care Anglia Ruskin University Chelmsford UK; ^2^ Department of Psychology, Institute of Psychiatry Psychology & Neuroscience King's College London London UK; ^3^ Bethlem Royal Hospital South London and Maudsley NHS Foundation Trust Beckenham UK

**Keywords:** chronic illness, identity, long‐term conditions, mental health, young adults

## Abstract

**Background:**

It has been suggested that the mental health impacts of living with long‐term conditions are greater in young adulthood compared to older adulthood, due to greater disruption to identity and routine life events.

**Objectives:**

To explore the impact of living with long‐term conditions in young adulthood on mental health and identity, and what helps living well with these conditions.

**Methods:**

Fifteen in‐depth interviews with young adults with various conditions were conducted and analysed thematically.

**Results:**

Themes related to the impacts on mental health and identity include the following: negative mood and depression; anxiety and fear for the future; and identity as ‘ill’/abnormal compared to former self and ‘normal’ others. Themes related to suggestions for addressing negative impacts include the following: promotion of positive thinking; support reaching acceptance with altered identity and limitations (through stages of denial, anger, depression, then acceptance); and more professional mental health support.

**Discussion:**

In order to promote mental health and a positive sense of self/identity, young adults with long‐term conditions should be offered advice and support on positive thinking; the long and difficult process of reconstructing identity; and reaching acceptance. This is particularly important for young adults for whom the identity reconstruction process is more complex and psychologically damaging than for older adults, as this life stage is associated with health/vitality and illness represents a shift from a perceived normal trajectory to one that appears and feels abnormal.

## 1 BACKGROUND

A long‐term condition (LTC), or chronic illness, is any on‐going health condition that can be controlled but not cured and requires individuals to self‐manage complex symptoms and treatments.^1^ Self‐management refers to the ability to manage symptoms, treatments, lifestyle changes and psychosocial consequences of an LTC and is considered important in improving health and well‐being outcomes. When it comes to defining young adulthood, the age range varies according to the chosen perspective. We have chosen to adhere to Erikson's stages of psychosocial development,^2^, ^3^ whereby young adulthood comprises ages 18‐40. Young adulthood is considered to be the healthiest time of life, comprising the peak years of life. Expected milestones include obtaining independence, forming romantic relationships, establishing a career and starting a family.^4^, ^5^ However, young adults with LTCs may not reach, or be delayed in reaching, such milestones.

Whilst the likelihood of having LTCs increases with age, there is a significant proportion of young adults with LTCs (15% of adults aged 20‐29 and 20% aged 30‐39)^1^ and their experiences and management strategies are under‐researched compared to other age groups. There is growing consensus that age‐related needs are more important than diagnosis in the promotion of self‐management (eg Ref.^6^) and that age‐specific interventions are likely to be better received by young adults (eg Ref.^7^). However, little is known about the experiences and needs of young adults with LTCs and there is a lack of age‐specific self‐management interventions. This paper reports a study which explores the experience of living with a LTC in young adulthood and what helps living well with LTCs at this age.

### 1.1 Impact of living with LTCs

The impacts of living with LTCs have been identified predominantly through research with older populations. Research has revealed that kidney disease,^8^ diabetes^9^ and various conditions^10^, ^11^ are associated with depression, anxiety and fear for the future. One of the postulated reasons for these negative mental health impacts concerns the change in identity that results from LTCs. Individuals with LTCs can experience a loss of their former self (if not diagnosed at birth/infancy) leading to the creation of a new identity with new restrictions which they then need to come to terms with. This has been outlined by research with primarily older adults (eg Refs^9^, ^11^, ^12^, ^13^).

The importance of acceptance for improved mental health has been described. Across studies with individuals of varying ages, acceptance has been described as being at peace with the limitations and losses associated with the condition, acceptance of the condition as part of your identity, and being able to appreciate and recognize the life lessons learnt. Acceptance has been described as a gradual and difficult on‐going process requiring time and reflection, and has been emphasized as important for living well with LTCs.^11^, ^14^, ^15^, ^16^, ^17^ Acceptance has also been found to be associated with better emotional functioning amongst young adults specifically.^18^, ^19^

### 1.2 Young adults with LTCs

A few studies have been conducted with young adults (with chosen age ranges of young adulthood varying between 14 and 45). Studies have found that cystic fibrosis,^14^ congenital heart disease^20^ and diabetes^21^ are associated with anxiety, depression, fear and worry; and that young adults with various LTCs are statistically more likely to experience anxiety and/or depression than healthy participants (eg Ref.^22^).

The aforementioned identity reconstruction process has been argued to be more complex and psychologically damaging for younger adults than for older adults. Hunter et al^23^ found that young people with venous thromboembolism reported a greater impact on their life from the condition than older adults, including greater mental health difficulties. The authors attributed this to the significant challenges younger adults face in integrating their experience of the condition into their identity, which occurs out of developmental context. They also concluded that young adults are likely to experience more disruption to routine life events such as occupation, pregnancy, travel, planning for the future and engagement in sport. Other studies exclusively with young adults have also touched on this topic. Studies have found that young adults with specific individual conditions have a sense of being different; struggle with a shift from an identity of healthy to sick; and feel that the challenges they face are not age‐appropriate.^24^, ^25^, ^26^, ^27^

Relevant to the impact of LTCs on identity is the concept of biographical disruption.^28^, ^29^ For those with LTCs, there is a biographical disruption between the individual's definition of themselves with regard to the past, the present and the future. The concept of biographical work involves redefining one's identity through a process of discovering which aspects of the identity have been lost (leading to feelings of loss and grief), which aspects remain, and which new aspects have been added.^30^, ^31^ The biographical work also includes a process of coming to terms with the situation, with the illness becoming an integrated part of the identity.^32^ This approach acknowledges that being diagnosed with a LTC in young adulthood forces rapid changes to personal identity, as this life stage is associated with health and vitality across cultures^33^, ^34^ and a LTC implies premature ageing that represents a shift from a ‘normal’ trajectory, to one that appears and feels abnormal.^28^, ^35^

### 1.3 Aims and objectives

In summary, research has demonstrated a negative impact from LTCs on mental health and sense of self and identity; and these impacts are interrelated. Most of the research has been conducted with older adults and/or focusing on individual conditions (such as diabetes, for example). However, qualitative research is needed into the experiences of young adults across conditions in relation to their mental health and identity in order to inform age‐appropriate (in addition to condition‐specific) strategies/interventions for living well with LTCs. This is particularly important given the argument that identity reconstruction is particularly psychologically distressing for younger adults, and therefore, reaching acceptance can be a more gradual and difficult, yet integral, process. The aims of this study were to explore the experience of living with LTCs in young adulthood and what helps living well with LTCs. The current article focuses on experiences and suggestions for living well related to mental health and identity, whilst other papers will focus on social life and relationships.

## 2 METHODS

### 2.1 Design

In‐depth semi‐structured one‐to‐one interviews were conducted with young adults with a range of LTCs. Thematic analysis^36^ was used to inductively identify themes about the experience of being a young adult with LTCs and suggestions for living well in this context. The methods are reported adhering to the consolidated criteria for reporting qualitative studies.^37^

### 2.2 Participants

The eligibility criteria were ages 18‐40, English speaking, and currently living with one or more LTCs for a minimum of six months. Individuals were not eligible to participate if they had extensive cognitive impairment affecting their capacity to independently care for themselves. Participants comprised 10 females and five males, aged 19‐39. The majority lived with more than one LTC (see Table 1). The mean time since diagnosis was 9.6 years, and age at diagnosis ranged from three to 35 (mean age of diagnosis: 18.7 years).

**Table 1:** Participant demographics

Pseudonym	Gender	Age	Employment status	LTC/s	Time since diagnosis
Ruth	Female	30	Part‐time employed	Chronic fatigue syndrome	6 y
				Asthma	2 y
				Fibromyalgia	<6 mo
Rebecca	Female	24	Part‐time employed	Microprolactinoma (benign tumour on pituitary gland)	4 y
				Chronic fatigue	4 y
				Connective tissue autoimmune disease	<6 mo
John	Male	39	Part‐time self‐employed	Post‐traumatic stress disorder	4 y
				Eating disorder	Not specified (‘for years’)
Anna	Female	25	Full‐time employed	Crohn's disease	10 y
Elizabeth	Female	29	Part‐time employed	Chronic fatigue syndrome	2 y
				Fibromyalgia	2 y
				Irritable bowel syndrome	2 y
Samantha	Female	27	Full‐time self‐employed	Systemic lupus	4 y
				Lupus nephritis	4 y
				Chronic migraines	‘Since childhood’
				Depression	6 y
Ella	Female	29	Full‐time employed	Thyroid disease (and removal)	18 y
				Chronic fatigue	18 y
James	Male	32	Full‐time postgraduate student	Irritable bowel syndrome (IBS‐D)	10 y
				Social anxiety disorder	10 y
Matt	Male	26	Full‐time undergraduate student	Chronic fatigue syndrome (later changed to below diagnosis)	14 y
				Benign brain cyst	7 y
Chloe	Female	31	Full‐time employed	Chronic fatigue syndrome	12 y
Zoe	Female	19	Full‐time undergraduate student	Type one diabetes	12 y
Luke	Male	32	Part‐time employed	Neuropathy	29 y
Alice	Female	20	Full‐time undergraduate student	Asthma	4 y
				Depression	4 y
				Anxiety	4 y
Tom	Male	31	Full‐time employed	Multiple sclerosis (relapse and remitting)	7 y
				Chronic fatigue	7 y
				Depression	7 y
Emily	Female	26	Part‐time employed	Grave's disease (and thyroid removal)	4 y
				Depression (later attributed to above)	6 y
				Anxiety	6 y
		Mean: 28			Mean: 9.6 y

Note

Some participants had a primary diagnosis of chronic fatigue syndrome whilst others experienced chronic fatigue as a result of their primary diagnosis. There were also a number of participants who reported experiencing clinically diagnosed depression and/or anxiety (as indicated in table) but a further two reported undiagnosed/self‐reported anxiety and/or depression.

### 2.3 Procedure

Ethical approval was obtained from the Anglia Ruskin University Faculty Research Ethics Panel. We recruited from the East of England through UK‐based charities for specific LTCs, social media and the Anglia Ruskin University website. Interviews, conducted by the first author, took place July‐October 2016 either at one of the Anglia Ruskin University campuses or in a community venue more convenient for the participant. Interviews lasted between 31 minutes and 1 hour 31 minutes. Participants provided written informed consent prior to taking part. A flexible topic guide (see Appendix 1) was devised which explored the experiences of living with the condition/s, impact of the condition/s on various aspects of life and what helps to live well with the condition/s. The findings related to mental health and identity are explored here. As interviews involved discussion of sensitive topics, if participants became upset the interviewer offered to stop the interview, offered a break and/or skipping a question. Interviews were digitally voice‐recorded and transcribed verbatim for data analysis. Participant names and identifying information were replaced with pseudonyms.

### 2.4 Data analysis

The data analysis was guided by Braun and Clarke's thematic analysis framework^36^ to inductively identify themes and theories about the experience of being a young adult with one or more LTCs, and suggestions for living well in this context. Themes were drawn inductively from the data, and theoretical concepts have not been imposed on the data, only those that coincide with the data have been used. An inductive approach was chosen as this is an exploratory study looking at an emerging area (focusing on young adults with various LTCs) which aims to inform future self‐management interventions and strategies. Data analysis was conducted primarily by the first author, who sequentially read through each of the transcripts, making notes about potential themes, the key experiences and suggestions of each participant, and personal reflections on how the data aligned with and differed from the researchers' own experiences of living with LTCs (see ‘Reflexivity’). The first author then re‐read each transcript, ensuring that key experiences and suggestions had not been missed. Initial codes were discussed with the second author, and then, codes were grouped into themes and sub‐themes by the first author. A review of themes then took place by both authors, with the first author re‐reading the transcripts once more, and the second author reading the transcripts to review the themes in order to ensure rigour. Once a consensus had been reached, themes were defined and labelled and agreed by both authors. Participant quotes were used to illustrate each theme.

### 2.5 Reflexivity

Both authors have quantitative and qualitative psychology backgrounds, with the second author also training as a Clinical Psychologist. Both authors are themselves young adults living with LTCs. The authors engaged in reflexive practices such as keeping a reflexive journal and consulting with academic mentors through all stages of the research (see Appendix 2 for extracts from the first author's reflexive journal during the data collection phase).

## 3 FINDINGS

Six sub‐themes were identified in relation to the impact of LTCs and five in relation to suggestions for living well with the conditions, related to the overarching theme of mental health and identity. The core sub‐themes are described below and illustrated using quotes. For details on the remaining sub‐themes, please contact the authors.

### 3.1 Negative mood and depression

Twelve of the young adults reported feeling down, fed up, upset, angry, frustrated and overwhelmed, as a result of their LTC/s. For example:

…you get in a bit of a funk, and you’re kind of like, “Ugh, I wish I could get up, I wish I could do this, I wish I could do that”…you can feel really down… (“Ruth”; a pseudonym, 6 years since diagnosis)

I…get quite bad bouts of irritability and feeling down…there are days when I just think, “Do you know what? I’m really f*****g fed up. I’ve had enough…” (“Elizabeth”, 2 years since diagnosis)

For some, they experienced clinically diagnosed depression and suicidal thoughts as a result of the difficulties in living with LTCs, for example:

I've had some really low days…where I've really felt like it’s not worth it…I questioned, “What’s the point in living?”…For years I’ve swung between wanting to [commit suicide] and not… (“John”, 4 years since diagnosis)

I mean I’m not sure how I would be if I didn’t have [my daughter]…because my depression, it creeps up on me…if I didn’t have her…I’d probably be dead by now because I would have just given up on everything. (“Samantha”, 4 years since diagnosis)

It affected me more than I thought it would mentally…it’s a physical issue but the effect it had on my mental health…is quite surprising… (“Emily”, 6 years since diagnosis)

For some, the sadness, depression, anger and frustration were particularly pronounced at the diagnosis stage, for example:

[After diagnosis] I went on antidepressants for about a year because…it was such a shock…at that initial diagnosis you just kind of think, “Is this all my life has amounted to?”…”is it worth going on?” I’m really positive but when something hits you that hard…you get seriously depressed. (“Tom”, 7 years since diagnosis)

A lack of mental health support (aside from antidepressants) from diagnosis and throughout their journey of living with the condition/s to the present day was also identified:

…there isn’t that support, there isn’t one place you can go to…You have to jump through so many different hoops to actually get the help that you may need…I recently…had a bad time mentally…so I went to the doctors and there’s so many hoops to jump over…I’ve [still] not had any counselling or therapy…getting any sort of help is so difficult it’s just, “Here take this medication and you know if you feel like you’re going to kill yourself then you give us a call. But we’re only open 9am until 5pm”. (“Ella”, 18 years since diagnosis)

My doctor is fairly useless…you say, “I’m really struggling”, and they go…”Well take some tablets”. “That isn’t what I came for…”…their quickest solution is to write out a prescription and send you on your way…not very helpful…I would like to be listened to, rather than, “That’s the only option”. (“John”, 4 years since diagnosis)

### 3.2 Anxiety and fear for the future

Eight of the young adults described experiencing anxiety (whether clinically diagnosed or self‐reported) and fear for the future as a result of living with their LTCs. For example:

…I have these blind panic moments…”Oh my God, what’s happening?” Which doesn’t help to my overall wellbeing. (“Elizabeth”, 2 years since diagnosis)

…I’m paranoid that it’s going to flare up…it’s always in the back of your mind, eating away at you… (“James”, 10 years since diagnosis)

I think one thing I hope for is that I’ll be able to manage until the end of my life because I don’t know what kind of a turn it might take… (“Luke”, 29 years since diagnosis)

As with depression, feelings of anxiety and fear were also reported to be most pronounced at diagnosis:

…it was quite scary, not knowing what the future held…when I first got diagnosed I used to be scared of going out anywhere because the condition was so unpredictable…. (“Anna”, 10 years since diagnosis)

At the time [of diagnosis] I was pregnant and my first thought was, “Oh my God, what’s going to happen to my baby?” at which case she was then born 11 weeks premature because my kidneys failed…young mothers that get diagnosed through pregnancy they don’t know how to kind of deal with being pregnant and having this illness all of a sudden…and dealing with emotions of having to face that your baby may not survive, that you may not survive…I didn’t get any kind of emotional support through that…that’s what I really needed… (“Samantha”, 4 years since diagnosis)

### 3.3 Identity as ‘ill’/ ‘abnormal’: compared to former self and ‘normal’ others

Negative impacts on sense of self and identity were widely reported. Nine participants made comparisons between their former ‘well self’ and their current ‘ill self’. Some longed for their ‘well self’, some postulated what they would be doing or how their identity would be different if they hadn't become ill, some noticed changes in their personality, and some longed for particular activities they could no longer partake in which were previously valued parts of their identity.

I remember life before [being ill] and so not being able to do everything that I’ve enjoyed doing previously‐ I know it’s been six years now but I can still remember that time before. I’d love to be able to stay up, I was at uni then, so I did stay up and do loads, it was a good time. But I wouldn’t cope with university now and it’s hard knowing that I will probably never get back to that. I don’t like that…I’m not the person I wish I was, and how I was prior to having this. It’s part of me, though. I come as an ill person… (“Ruth”, 6 years since diagnosis)

Four participants made reference to their previous sporty/athletic selves and saw this as forming part of their pre‐illness identity which they had now lost, for example:

…the biggest thing I can’t do is play sports and that’s quite frustrating because I used to play quite a lot of sports…and I enjoyed it…I just can’t do it. I just haven’t got the energy. (“Chloe”, 12 years since diagnosis)

One participant described this as grieving for his former self, and grieving for the loss of part of his identity:

…you grieve for the previous person you were…it’s quite horrible…when I went blind for two weeks and where I couldn’t walk for three months and after being very active, I was playing hockey…and I was very sporty…so basically that just went…so you kind of grieve that and you just think, “Is it worth going on?”… (“Tom”, 7 years since diagnosis)

Two participants specifically referenced a sense of loss at no longer being able to drink alcohol as a result of their LTC/s which impacted on their sense of identity, for example:

…I now don’t touch alcohol because the slightest little drink, the next day I am completely done…I have had to obviously leave those sorts of things completely alone and the drinking before…was quite a large part of my lifestyle. (“Samantha”, 4 years since diagnosis)

Two participants made comparisons between their old ‘well self’ whom they viewed as intelligent, and their new ‘ill self’ whom they saw as less intelligent. This perceived loss of intelligence led to negative views of self and feelings that their potential was being constrained:

I was very, very intelligent and I no longer feel like I am intelligent because there are days when I struggle to string a sentence together…that really upsets me…I feel I have so much potential and it’s being smothered… (“Elizabeth”, 2 years since diagnosis)

The notion of being ‘abnormal’ compared to ‘normal’ others was also described by eight participants. Some references were made to feeling ‘abnormal’ in comparison with others in general, for example:

…I’m not normal and I’m not capable of normal things. (“Elizabeth”, 2 years since diagnosis)

You see everyone else…It’s easy to assume everyone else is living a perfect life, that they’re not worried about anything. So you’re like, “Oh, why can’t I be more like them?” (“James”, 10 years since diagnosis)

When Ruth was asked ‘what would living as well as you can with your condition look like to you?’, she described being able to fulfil certain tasks and life goals and summed it up as ‘being able to function as a normal person’. Zoe also reported previously being in denial of being ill and wanting to ‘be like everybody else’ and even stopped taking medication which had negative implications for her health:

…I…got in my head that it wasn't real and I didn't want it. If I didn't take my insulin, if I didn't do my test then it wouldn't be there because I'd just be like everyone else. That messed up my blood sugars… (“Zoe”, 12 years since diagnosis)

However, over time had reframed what she considers ‘normal’ as a way of adapting:

That's something that when I was younger and in that mind‐set that no I wasn't diabetic, like I didn't want to do my tests and stuff, that was the wrong way to go about it. That was, “I can be normal if I'm not doing the tests and stuff”. No it's that, “I can be normal if I *do* my tests and stuff. Then I can do what everyone else is doing…I think that was a big thing for me, finally realising that and being like, “Yes I'm not normal but what's normal”. (“Zoe”, 12 years since diagnosis)

Four participants made a comparison between themselves as ‘abnormal’ compared to other ‘normal’ young adults, feeling that their life and their identity were opposing to expectations for young adults:

…you always have a feeling of what you want at this age…to be married, with children…and it hasn’t happened. I blame my condition on that… (“Ella”, 18 years since diagnosis)

…usually at this stage, like with my mum and brother…my mum would have taken much more of a backseat…he was enjoying life like a 24‐year‐old should. But she can’t do that with me… (“Rebecca”, 4 years since diagnosis)

I felt like I had a condition that’s something that older people get…when I got diagnosed…I was 22. I thought, “Why is this happening to me at this age?”…It made me feel‐ I don’t know if “broken” is the right word, but certainly different, not like everyone else. Everyone else is, “Oh, you don’t have to worry about medical things until you’re much older.” But, for me, it was like, “Oh, I already have to think about this”…I think when you’re older, like a senior citizen, it’s, kind of, expected that you’re not in good health. So, if something happens, people are all, “Oh, he’s old so there must be something wrong.” Whereas, if you’re younger and something’s happening, people just think “You’re strange, something weird’s happening there.” (“James”, 10 years since diagnosis)

Two participants highlighted the difference between the reality of their life as a young adult and the culturally accepted ‘norm’ of young adulthood, by comparing their young adult selves to the lives of older adults for whom illness is more expected and accepted as normal, for example:

…at one point I was taking like 24 pills a day, which was really strange, because my grandma’s like 80, and really old and got lots of issues and she was like, “I only take 10”. So I like, thanks. That makes me feel so good…that makes you feel very weird as a 24 year old. (“Emily”, 6 years since diagnosis)

### 3.4 Positive additions to self and identity

Despite everyone reporting some negative impact from their LTCs on their sense of self and identity, seven participants reported some positive impacts: becoming more resilient, compassionate, empathetic, mature and motivated, for example:

…it has also made me a stronger person…it’s made me a better, more caring and considerate person…it’s made me want to help other people…it’s made me the person I am. (“Ella”, 18 years since diagnosis)

…it made me mature quicker…I feel like I appreciate other people and their struggles a lot more, I can empathise with what people are going through. (“Anna”, 10 years since diagnosis)

### 3.5 What helps to live well with LTCs?

#### 3.5.1 Promotion of positive thinking

Nine participants emphasized the importance of positive thinking and not dwelling on the negatives/limitations. Suggestions included focusing on the things you can do, making the most of your good days, planning fun things to do, remembering some of the positive impacts of the condition/s and celebrating small achievements.

…live your life to the maximum extent that your condition will allow you to do, and plan really nice things for you to do when you are well. It’s really important that you don’t spend your days that you are well just mulling over the fact that you've got a condition. And just try and think about the positives of it, there aren’t many, but it makes you more resilient… (“Rebecca”, 4 years since diagnosis)

I try and look at things as positively as I can…I always try and put a positive on it. It has been horrible…but I’m almost glad it turned out the way it did… I do think that getting ill, in some respects, worked out quite well. I’ve got a job I love…I’ve made friends I would never have made, I’ve done a course I would never have done…it’s definitely had positives and…I make a mental point of trying to focus on those a lot more… (“Emily”, 6 years since diagnosis)

Life is too short…I know that eventually…this is what’s going to kill me. I don’t know how long I’ve got, so why not make the most of it?…I’ve got to try and remain positive and…do as much as I can with the good days. (“Samantha”, 4 years since diagnosis)

#### 3.5.2 Accept yourself, who you are and your limitations

Nine participants mentioned that reaching a point of acceptance about the condition, about your limitations and about your changed identity and lifestyle is essential to living well with a LTC. This was described as a process that had to be learnt over time and participants described going through various stages before reaching acceptance (eg denial, anger, depression).

Sometimes I need a reminder and I’ll get really cross with myself and say…”you need to do this, you need to do that”, and I’ve learned the hard way that I need to…say “No, you’ve got your limitations and you just need to accept it”. (“Elizabeth”, 2 years since diagnosis)

…something that I finally realised, it's just part of me. I think a huge part of people getting used to LTCs is you kind of just have to accept that it is a part of you…Until you've accepted yourself, and that it's a part of you, you're not going to move on and you're really going to struggle. (“Zoe”, 12 years since diagnosis)

#### 3.5.3 More professional mental health support

More mental health support, such as counselling or therapy, was suggested by five participants, with emphasis on early intervention soon after diagnosis, for example:

…I think [counselling] should be available. Anyone, especially at a young age, who goes through something like that should have…support and someone to speak to…a mental health professional…Any young adult who goes through something like this it’s going to take a very, very, very strong individual not to suffer from it. And I think that [mental health professionals] should get involved much earlier on. (“Ella”, 18 years since diagnosis)

## 4 DISCUSSION

The aims of this study were to explore the experience of living with LTCs in young adulthood, and what helps to live well with LTCs. The overall aim is to inform future strategies (that are not condition‐specific) to help young adults live well with LTCs, with the aim of improving well‐being, and promoting a positive sense of self and identity. Fifteen young adults aged 19‐39 with a range of LTCs were interviewed, and a number of similarities across conditions were found in relation to the experienced impact on mental health and identity, and the suggestions made to address these impacts. The following core themes related to the impacts of the conditions were discussed as follows: negative mood and depression; anxiety and fear for the future; identity as ‘ill’/‘abnormal’ compared to former self and ‘normal’ others; and some positive impacts on self and identity. Key themes related to the suggestions for living well with the conditions, which address the negative impacts, were as follows: promotion of positive thinking; acceptance of self and limitations; and more professional mental health support. The current research findings add to the sparse qualitative research with young adults which explore similarities in experiences and management strategies across conditions. Some of the findings align with research with older populations; however, the unique and intensified needs of young adults with LTCs are highlighted, as explored further below.

Twelve out of 15 participants described experiencing negative mood and in some cases clinically diagnosed depression and suicidal thoughts as a result of their condition/s, and eight reported experiencing anxiety and fear for the future. This complements and expands on earlier findings with predominantly older adults and/or amongst individuals with specific individual LTCs.^8^, ^9^, ^10^, ^11^, ^14^, ^20^, ^21^ Nine out of 15 participants made comparisons between their former ‘well’/’healthy’ self and their current ‘ill’/‘sick’ self, with longing for their past self and grieving lost parts of their identity (although it is important to note that this sub‐theme is only applicable to young adults diagnosed from birth/infancy, perhaps excluding for example congenital illnesses such as cystic fibrosis, where there is no prior period of ‘wellness’ or perceived ‘normality’^38^). Over time individuals reconstructed their identity and described having brought themselves to a point of acceptance. This has been found with older adults with LTCs (eg Refs^11^, ^12^) and with young adults with specific conditions such as diabetes^24^ and lupus.^26^ Despite not having a prior period of wellness/'normality’, the importance of acceptance has also been identified amongst young adults with cystic fibrosis.^14^ The current study suggests that this is the case for young adults across a range of LTCs.

A particularly unique finding for young adults, as opposed to older adults, is that eight of those in the present study also made comparisons between ‘normal’ others and their ‘abnormal’ self. In some cases, their perceived abnormality was in relation to other people in general, but for many it was in comparison with other ‘normal’ young adults. ‘Normal’ for young adulthood was seen as meeting societal expectations for this life stage, such as being energetic and healthy and achieving milestones such as marriage, having children, establishing a career and gaining independence from parents. This was also highlighted through comparisons to older adults for whom ill health was viewed as more expected/accepted. It has been postulated that this added challenge to identity reconstruction exacerbates the negative mental health implications from LTCs for young adults.^23^, ^39^^.^

The present findings fit well with the concept of biographical disruption^28^ which encompasses a disruption in the individual's pre‐illness identity and their current ‘ill’ identity. As fitting with descriptions by the current participants, biographical work comprises a loss of aspects of the former identity which leads to feelings of loss and grief but also the addition of new aspects which can be negative and positive. This process of identity reconstruction is particularly challenging for young adults as this life stage is culturally associated with health/vitality and experiencing illness marks a biographical shift from a perceived normal trajectory. Therefore, the ability to integrate the illness into part of the identity, and to accept this altered identity, is both particularly challenging and important for young adults and an important focus for future self‐management interventions.

One of the most dominant suggestions made for helping young adults live well with LTCs was to reach a point of acceptance. This was described as a process that had to be learnt over time and participants described going through various stages before reaching acceptance (eg denial, anger, depression). This fits with Kubler‐Ross's model of grief,^40^ whereby people are described as moving through five linear stages of emotional adjustment: denial, anger, bargaining, depression and acceptance. Indeed, participants did largely describe the same linear order of stages in coming to terms with their LTC/s—particularly amongst those who had been diagnosed for a longer period of time who reflected back on their feelings over the years since diagnosis. This also expands on previous research which has identified reaching acceptance of new/altered identities as important for mental health, and was also seen as a long and difficult process that required time and reflection (eg Refs^11^, ^16^). The other suggestions made by participants in the present study are less present in the existing literature, although some reference has previously been made to the value of positive thinking (eg Refs^8^, ^11^, ^17^, ^23^, ^26^).

Some limitations of the study warrant consideration. Firstly, more females than males self‐selected to take part. Secondly, the current sample represents a relatively ‘healthy’ group of individuals in that all of them, at the time of interview, were able to work or study in some capacity. Additionally, all participants were from a white ethnic background (with all but one from a white British background) again due to the nature of those who self‐selected to take part. Thus, findings from this study may not be representative of the wider population of young adults living with LTCs. It is also important to consider the variety of time since diagnosis ranging from two to 29 years (although it is worth noting that a number of participants reported experiencing symptoms long before receiving a diagnosis). It is acknowledged that the length of time a participant has been living with their LTC is important to their personal narrative and that time since diagnosis would have impacted on reported experiences. However, it was considered important to include participants with a variety of conditions and experiences in order to ascertain whether there are similar experiences across individuals which could inform a wide‐reaching strategy to help young adults live well with LTCs.

In summary**,** as in older adulthood, living with LTCs in young adulthood seems to be associated with negative mood (in some cases clinically diagnosed depression and suicidal thoughts), anxiety, fear for the future, upsetting comparisons between current and former selves, and having to reach a point of acceptance with their altered identity. However, a particularly unique finding for young adults is the presence of distressing comparisons between ‘normal’ others and ‘abnormal’ selves due to societal expectations of young adulthood, with illness perceived as more expected and socially accepted in older adulthood. This is thought to exacerbate the negative mental health implications for young adults, compared to older adults. In order to address this, young adults with LTCs are wanting more professional mental health support and identify several areas that could be targeted in psychological treatment: promotion of positive thinking; support with the long and difficult process of reconstructing identity (through stages of denial, anger and depression); and reaching acceptance with the new identity (including acceptance of limitations and losses, and recognition of the positives and life lessons from the condition/s). Whilst medical management of LTCs is a vital component of self‐management, when asked what can help them live well with LTCs, young adults focused on loss of normality, change in identity, coping with the mental health implications of their LTCs and the importance of reaching acceptance with their altered identity and life trajectory. The findings highlight the importance of multi‐disciplinary team working to ensure physical and psychological needs are addressed for young adults with LTCs, with mental health professionals playing an important role to ensure that psychological aspects are addressed in all aspects of treatment. As part of this, policymakers should be implementing strategies which will enable health‐care services to deliver better access to professional mental health support, soon after diagnosis, which is targeted to the unique and significant struggles young adults with LTCs face. It is essential as a next step to research the current barriers to accessing mental health support and ways to address this; talking to mental health professionals about their experience and knowledge of providing psychological therapies to young adults with LTCs; exploring in collaboration with health‐care providers and patients what a targeted mental health intervention for young adults with LTCs may look like; and the feasibility of rolling‐out such a service. In the meantime, this study has provided an essential first step in identifying the need for, and current lack of, sufficient professional mental health support for young adults with LTCs and what aspects this support should encompass.

## CONFLICT OF INTEREST

The authors declare that there is no conflict of interest.

## APPENDIX 1

### INTERVIEW QUESTIONS

*Background questions*

1. Can you tell me a bit about your long‐term condition or conditions?
2. Can you tell me about your initial feelings of being diagnosed with a long‐term condition?
3. Could you tell me a bit about your general experience of living with a long‐term condition?

*Impact on your life*

1. Have you had to make any adjustments to how you live your life in light of your condition? (If yes, how?)
2. What is the main limitation that your condition has on how you want to live your life?
3. Does your condition have an impact on your social and family life and if so how?
4. Does your condition impact on your ability to work and/or study and if so how?
5. Does your condition impact on any other aspect of your life?
6. Do you feel that your condition impacts on how you view yourself and if so how?
7. Do you feel that your condition impacts how others view you and if so how?

*What helps and what doesn't?*

1. What would living as well as you can with your condition look like to you?
2. What would help you to be able to live as well as you can with your condition?* Probes: what currently helps you? what else could help you?*
3. Do you feel that social media has any role to play in helping you to live well with your long‐term condition?
4. If you could give one piece of advice to another young adult living with a long‐term condition what would it be?
5. Do you think there is anything that would be helpful to young adults with a long‐term condition that isn't currently available?

*Final thoughts*

1. Is there anything else you would like to tell me about what it is like living with a long‐term condition?

## APPENDIX 2

### EXTRACTS FROM [THE FIRST AUTHOR'S] REFLEXIVE DIARY DURING DATA COLLECTION AND ANALYSIS

I could definitely relate to most of what “Ruth” said…I also used to frequently find myself longing for my former “well” self which wasn’t helpful—I have had to learn to stop this, grieve for my former self, and focus on the now and the positives about the now (including how I have grown and developed as a person because of my experiences). I agree with “Ruth” that celebrating small achievements and focusing on the positives is important when living with LTCs. And also not to compare ourselves to “well” others. A lot of what “Samantha” talked about was related to her being a mother, which I cannot yet relate to. It was very informative to hear from this perspective, and to consider the added challenges and benefits of having a young child whilst managing a LTC. I was particularly moved by the role that her daughter has played in helping her to “keep going” through her depression. I did relate to a number of other things that “Samantha” talked about…I too have noticed a change in my outlook/perspective over time as I have accepted my LTCs and adapted accordingly. Out of all of the interviews I related most to “Ella”—nearly everything she said I also experienced and felt. I too, in particular, have felt very driven and motivated academically (feeling the need to prove myself) because of my LTCs. I too worry about being perceived as flaky and unintelligent because of my fatigue and the fluctuating nature of my conditions. I don’t think I would have had the drive and determination to get a PhD if it wasn’t for my LTCs…Strangely despite all the negatives I also don’t think I would change having it as it has definitely shaped the person I am and changed me for the better in a lot of ways (…more sensitive, more resilient). As with “Ella” I too have found myself putting on a mask and bottling up my feelings and then experiencing mental health difficulties, and have also not had suitable mental health support. I have had more success when seeking this out than “Ella” has though…most recently having had an unhelpful experience with a CBT therapist who had very limited understanding of how my LTCs affect my mental health. “Zoe” was an incredibly inspiring young woman. I felt very encouraged and uplifted by the interview…I was really inspired by her outlook and maturity for her age (which I didn’t have at age 19). We had a number of similarities, despite having very different LTCs. It turned out that we both grew up in the same area…were both diagnosed at the same age and both went through a stage of depression in our teens. However, she has managed to reach a point of acceptance sooner than I managed to and she provided some useful advice to other young adults with LTCs. “Luke” was very friendly but appeared quite shy and slightly apprehensive about the interview and asked a few questions before we began. I reassured him that he didn’t have to answer any questions he did not wish to and explained the purpose of the research. I really admired Luke’s positive attitude towards his LTC, which has not always been the case for me. Luke did not express as many “negatives” as some other participants and seems to have gained a very admirable attitude towards his condition. Perhaps this is partly due to being diagnosed at such a young age (age 3)? He attributes it to his parent’s positive approach/attitude towards his condition that they instilled in him from a young age.
